# Energy Conversion Strategies for Wind Energy System: Electrical, Mechanical and Material Aspects

**DOI:** 10.3390/ma15031232

**Published:** 2022-02-07

**Authors:** Anudipta Chaudhuri, Rajkanya Datta, Muthuselvan Praveen Kumar, João Paulo Davim, Sumit Pramanik

**Affiliations:** 1 Functional and Biomaterials Engineering Lab, Department of Mechanical Engineering, Faculty of Engineering and Technology, SRM Institute of Science and Technology, Kattankulathur, Chennai 603203, India; anudiptac@gmail.com (A.C.); rajkanyadatta99@gmail.com (R.D.); mpk16199@gmail.com (M.P.K.); 2 Department of Mechanical Engineering, Campus of Santiago, University of Aveiro, 3810-193 Aveiro, Portugal; pdavim@ua.pt

**Keywords:** renewable energy, wind speed, wind turbine, power, power signal, torque, composite

## Abstract

Currently, about 22% of global electricity is being supplemented by different renewable sources. Wind energy is one of the most abundant forms of renewable energy available in the atmospheric environment due to different air-currents spread over the troposphere and stratosphere. The demand of modern wind energy conversion system (WECS) has increased to achieve a suitable alternate renewable energy source. In this paper, after a brief introduction, the classification of WECS is reviewed with attractive illustrations. The various mechanical materials and electrical components of WECS are discussed. The flow of power in WECS and its control strategies are also been described. The wind energy conversion is carried out with a suitable controlling mechanism for power grid integration. A maximum power-point tracking controller is an effective controlling method to extract the maximum possible power from the turbines. The present trends in WECS and the scope for improvement and future prospects are discussed. The materials used for both the blade and generator have been found to be key elements of wind turbines. Recycling of the polymer matrix composite materials are found to be a great threat to wind power plants, as well as to their supply chain industries.

## 1. Introduction

The demand of energy over the globe is unremittingly growing owing to the growing of population, energy consumption per capita and huge industrialization. The availability of conventional fossil fuels is limited [1]. Thus, energy crises in the future will be increasing more. Therefore, the use of renewable energy sources, such as solar [2], wind [3], biogas [4], ocean [5], tidal [6], and so on is emerging at tremendous rate. Among these, wind energy is one of the most promising sources. Wind energy has been used for a multitude of purposes for a millennium across the globe. From the use of sails in boats and rafts, to wind driven mills for grinding grains, wind energy has found its use throughout history [7]. 

### 1.1. A Brief History of Turbine Energy Conversion Technology

The early usage of turbines was found in the form of a carousel, like the vertical axis turbines in Persia used for pumping water and grinding food grains in very small scale, around the 10th century. Horizontal axis turbines were adopted in England, which had to be manually oriented according to the direction of the wind currents, thus giving it the name post mill [8]. However, experiments on large wind machines started during the years of World War II with the introduction of a turbine that was capable of generating approximately 1.25 MW, making way for modern day turbine and grid systems [9]. Thus, the demand of modern wind energy conversion systems has been realized. By 2012, the global power production from renewable resources exceeded 1470 GW, within which 282 GW was produced in grids powered by wind farms [10]. Energy consumption is the backbone necessary to meet the daily requirements of civilization. Starting from burning wood or charcoal as a means of thermal energy, the heater systems are currently used from homes to heavy duty boilers or furnaces in metal and iron smelting industries. As a by-product, the harmful effects of these non-renewable and carbon-based fossil fuels are multiple. Introduction of cleaner and renewable sources of energy such as tidal, solar, hydro, geothermal and wind have brought about immense changes in the realm of energy systems. Presently, about 22.5% or 0.225 fraction of global electricity is being supplemented by different renewable sources [11]. From 2012 to 2014 the global wind power capacity plunged from about 282 to 370 GW, which composes almost 0.4 fraction of the total renewal electricity, generated worldwide [12]. A recent study also reported that, the energy generated by renewable energy added to the global electricity about 70% or 0.7 fraction of the net capacity by 2017. The wind electricity alone surged by 52 GW, reaching to 539 GW in 2017 [11].

Wind energy has been the most abundant form of energy available in the atmospheric environment with varying air currents spread over the troposphere and stratosphere. Wind energy has been used for a multitude of purposes for thousands of years across the globe. From the use of sails in boats and rafts, to wind driven mills for grinding grains, wind energy has found its use throughout history. Wind energy has been a staple for transportation in oceans until as recently as the 19th century. The early usage of turbines was found in the form of carousel like vertical axis turbines in Persia for pumping water and grinding food grains in a very small scale, around the 10th century [13]. Horizontal axis turbines were adopted in England, which had to be manually oriented according to the direction of the wind currents, thus giving it the name post mill. Large scale harnessing of wind energy has been implemented since the 17th century in Europe [14]. These have paved the way for the more effective and useful utilization of wind in the 21st century, with modernization in design and optimization enabling us to store energy in the form of electricity, using wind turbines [15]. The wind energy conversion system (WECS) is an integrated system comprising of wind turbines, generators, mechanisms for control and an integrating method. The turbines are responsible for converting the power of wind into mechanical power, thus harnessing the kinetic energy from the wind. The efficiency of the turbines is a function of shaft power recorded at the rotor shaft and the power available in the flow of the wind stream [16]. The mechanical components are used chiefly for the conversion to mechanical power and transmission to electrical components of the system, as well as providing support to the moving components. The amount of power that can be strained from the wind current depends upon a multitude of factors such as density of wind, shape of turbine rotor, blade measurements, wind velocity, and so on. The high power WECS are low speed, high torque at the turbine. These turbines produce power of more than 1 MW where the speed of the turbine shaft is in the range of six to twenty rpm. However, the generator shaft is that of high speed and low torque, and thus, to couple it with the rotor shaft a gearbox is added [17]. For an effective reduction in torque, a multiple stage gearbox is used in high power grid-connected WECS. A planetary gear assembly ensures even more effective reduction phase. The electrical components of the WECS mainly comprise of a generator, a controller system coupled with anemometer to detect the speed of wind available to control the operation of the turbine.

### 1.2. History of Wind Turbine Technology

The invention of lead acid batteries by Camille Faure in 1881 provided a viable option for researchers to advance their studies for harnessing wind energy through turbines, since lead acid batteries were lighter and smaller. In the following years, prominent researchers such as Professor James Blyth, and Charles Brush successfully built some forms of horizontal axis turbines that were capable of charging up the accumulators available and illuminate a single mansion or real estate [18]. In the 1890s, a significant surge of wind turbine technology in Denmark resulted in a peak power capacity of 30 MW harnessed by more than 2500 windmills across the country [19,20]. This figure metamorphosed to each windmill to produce approximately 25 kW by the end of the first decade of 1900s, thus doubling the efficiency of turbine technology [21]. During this period, the number of blades on the turbines reduced significantly as lesser blades rotating faster proved to be more efficient than more blades rotating slowly. This was found by Danish researcher Poul La Cour. Eventually, research brought back the design of the vertical axis turbine system. The Darrieus wind turbine was the first among the vertical axis wind turbines (VAWTs) patented by Georges Jean Marie Darrieus, a French aeronautical engineer [22]. However, experiments on large wind machines started during the years of the Second World War with the introduction of a turbine that was capable of generating approximately 1.25 MW [18]. This build was concluded by an American engineer Palmer Cosslett Putnam along with the Morgan Smith Company (Ashaway, RI, USA) that manufactured hydroelectric turbines. This turbine was the first one to be connected to an electricity distribution grid due to its huge power production capacity. Research on wind energy conversion slowed down during the middle of twentieth century due to the feeling that there was less need for wind energy since exhaustible resources for energy were available on a large scale. Not until the end of the century was there much progress observed. In 1978, the first multi-megawatt turbine was created by the Tvind School [23]. The turbine had a novel wing construction, which was made with assistance from German aeronautic specialists [24]. Not only did this prove to be a landmark in the technological advancement of turbines, but it also stands as the oldest functioning wind turbine in history. National Aeronautics and Space Administration (NASA, Washington, USA) pioneered the turbine technologies with their research that included a series of turbines. This knowledge is incorporated into the design of the most recent turbines, such as composite blade materials, steel tube towers, variable-speed generators mounted on the tower along with dynamic components such as aerodynamics of the blades in horizontal axis turbines, pitch and span control mechanisms [25]. A large-scale variable drive train wind turbine capable of creating 3.2 MW was patented by NASA [26]. It was a two-bladed structure. Over the following decades, research was undertaken to improve the aerodynamics of rotor and turbines that have boosted the efficiency of wind energy systems. Uniform wind has yields of around 20–30% efficiency in modern day turbines, and more and more wind farms are being set up, increasing the global wind power capacity annually. The year 2012 marks a landmark with the global wind capacity clocking in at almost 300 GW worldwide, which balanced the cost of wind power to be $0.06/kWh, and is less than one-third the cost of electricity in the 1980s [27].

### 1.3. Review Outlook

The demand for the modern wind energy conversion system (WECS) has increased to achieve a suitable alternate renewable energy source. In this review paper, the various mechanical and electrical components of WECS are discussed. The flow of power in WECS and its control strategies are also described. We elaborate on wind energy conversion with a suitable controlling mechanism for power grid integration. A maximum power-point tracking controller has been found as an effective controlling method to extract the maximum possible power from the turbines. The present trends in WECS and the scope for improvement and future prospects are also discussed. The typical materials used for the major components are discussed with neat illustrations. This review article will make the researchers’ job of selecting the materials best suited for the blade and generator easier. 

## 2. Classification of Wind Energy Conversion Systems (WECSs)

The WECSs fall into various categories depending on different factors. The most common classification of WECS is the one based upon the axis of rotation of the system or rotational axis, turbine, power control, and rotational speed control criteria.

### 2.1. Rotational Axis

There are two basic axes of rotation possible for a turbine with respect to the ground, such as the horizontal axis wind turbine (HAWT), which rotates in an axis parallel to the ground, and the vertical axis wind turbine (VAWT), which rotates in an axis perpendicular to the ground. The function of both types of turbine systems is to produce kinetic energy through wind pressure generated by the turbine and convert this into electrical energy.

**Figure 1:**
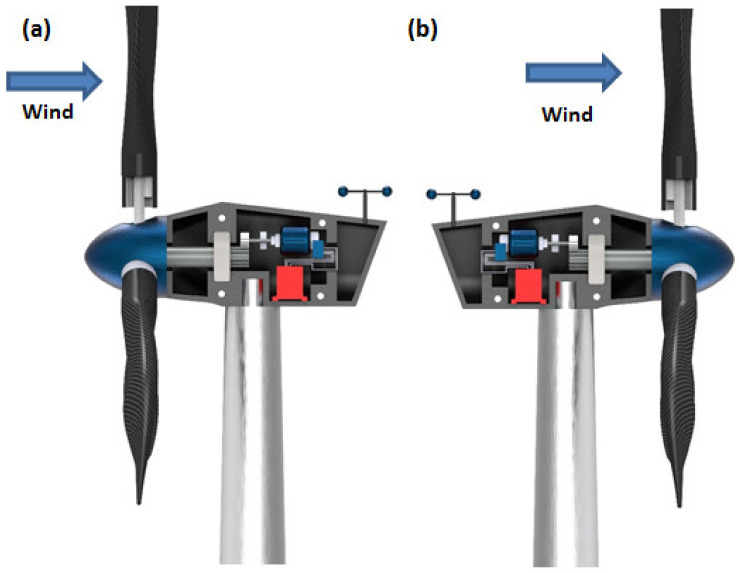
(**a**) Upwind HAWT (when wind is coming from the front side of the turbine blades); (**b**) Downwind HAWT (when wind is coming from the back side of the turbine blades).

- Horizontal Axis Wind Turbines: The shaft of the turbine and the shaft of the generator are placed parallel to the plane of the ground and mounted to a certain height to face the required wind speed depending on the geography. The elevated height of the turbine also facilitates the turbine to rotate with enough clearance to the ground. The mechanical components of the turbine are enclosed in a housing, which is shaped aerodynamically called a nacelle. The nacelle in this case is directly behind the rotor. According to the placement of the nacelle with respect to the turbine and tower, there are upwind and downwind HAWTs as shown in Figure 1 [28,29]. Upwind HAWTs have the turbine facing the direction from which the wind is blowing. The direction is adjusted automatically by the use of rudders. The downwind HAWTs have the turbine facing away from the direction of the wind flow. This type of HAWT faces the problem of vibrations in the blades due to the wind interacting with the nacelle first, making the wind turbulent. Commercial turbines incorporate the HAWT design mostly with three blades. However, two blade designs are also used on a very small scale. HAWTs demand high structural strength since the turbine, gearbox, generator, and so on are placed high above the ground.
- Vertical Axis Wind Turbines: This type of turbine has the turbine mounted on the shaft, which doubles up as the tower of the turbine structure and the rotor’s blades are vertically placed aerofoil sections. However, various designs of VAWTs are available, like Darrious and Savonious rotor turbines [30]. The installation and maintenance of these types of turbine are relatively cheaper and easier than other types. Since the turbine blades do not have to clear the distance between the ground and the hub of the rotor, the length of the tower can be kept short, which also helps to reduce the vibration of the shaft due to the rotating mass. However, the shaft does not need to hold the weight of the entire gearbox and control mechanisms as those components are placed at the base of the shaft near the ground. VAWTs are subjected to varying wind loads, differing with the height of the blade. Higher wind loads are experienced at the top of the turbine than the loads experienced at the bottom, rendering lower energy conversion efficiency. Thus, their uses for high-power application are restricted commercially.

### 2.2. Turbines

Turbines are also categorized based on their electrical output. The output power determines the size of the wind farm. Based on this, the prevailing turbine system technologies can be divided into three categories which are:

- Low Power turbines: These are turbine systems capable of producing on average a maximum of 30 kW. These systems find use in remote locations for household electricity demands and charging up of batteries. They are also used in the case of an emergency to reduce dependence on primary power sources;
- Medium Power Turbines: Turbines pushing out in the spectrum of 30 kW to 300 kW fall in this segment. However, they are mostly used to power up the homes in small localities. They are used along with other sources of renewable energy or other power storage devices;
- High Power Turbines: These refer to the systems where a large production of electricity is undertaken. These are incorporated in the large size wind farms that are associated to the power grids responsible for transporting the energy throughout cities.

### 2.3. Power Control

Power control ability plays an important part in the design of turbines, since they are completely dependent on wind flow for generating power. As mentioned earlier, each turbine is rated for producing certain power. This power gets overshot when the turbine is subjected to stronger winds than they were designed to withstand. While, this produces more power, there are possibilities of mechanical failures due to increased vibrations and resulting unbalanced loads. Thus, power production is restricted under increased wind speeds, which can be achieved by three controlling methods, namely passive stall, active stall and pitch controllers. The other control methods include grid side controller and phase lock loop controller. Besides, a crow bar circuit is also used to act as protection for the whole electrical circuitry [31]. To obtain optimal maximum energy from the wind resource, different optimization techniques are utilized. These are done by modeling the different components of the WECS and simulating the results.

- A passive stall controller of WECS controls the input power of the wind by virtue of the aerodynamics of the blades and does not include sensors or actuators to stop the motion of the blades. In such WECS, the blades are fixed so that there is no relative motion with the hub, generally incorporating bolted or riveted connection. The design poses a stall when the wind flowing through the rotor exceeds a certain speed. This takes place since the first blade that comes in contact with the wind turns the flow into a turbulent one and the other blades continue to rotate within the wake of the first blade, thus significantly reducing the lift force experienced by those blades, stalling the turbine. Mostly used in fixed type fixed speed WECS, these are the cheapest and simpler designs than other types of WECS;
- An active stall control system is similar to the passive system; however, it uses sensors to predict the wind speeds and changes the pitch of the blades by means of motors or hydraulic actuators to interfere with the wind in a certain manner that induces a high turbulence and thus creating a stall. Active stall control systems are observed in high power turbines;
- Another type of WECS in this category uses similar mechanism as the active stall-controlled design, but the action is slightly different. Unlike the active stall controller, the pitch controller changes the pitch of the blades so that they don’t produce lift by reducing interaction with the flow of wind. This is different than the previous type which depended on increased turbulence, which still utilizes aerodynamics of the blade. This control system produces faster response times and thus, it is used extensively at present in the high-power turbine systems.

### 2.4. Rotational Speed Control Criteria

The criteria of rotational speed control classify the grid connected with wind energy conversion devices into fixed- and variable- speed systems. Here, tip speed ratio is a required concept to explain the classification effectively. In a wind turbine, the tip speed ratio is defined as the ratio of tangential velocity of the blade-tip to that of the wind driving speed of the turbine. When the turbine of radius (*r_T_*) is rotating at an angular velocity (*ω_T_* rad/s) and the velocity of wind (*ν_w_*) acting upon it, the tip speed ratio (*λ*) can be expressed by Equation (1) [28], which identifies the relation between the *λ* and the design parameters of the turbine.

(1) $$\lambda =\frac{Tangential\;speed\;of\;blade}{Speed\;of\;Wind}=\frac{\omega_{T}\;r_{T}}{v_{w}}$$

- Fixed Speed WECS: This type of turbine system composes the first generation of commercially set-up grid connected turbines. They are well proven over the ages and have reliable and simple construction. However, the simple nature of these turbines makes them operable only at a certain rated wind velocity, thus the name fixed-speed WECS. Winds at a faster velocity reduce the power coefficient (*C_p_*) of the turbine. The *C_p_* of a turbine is a unitless constant, particular to the design of the turbine. It is a ratio of power captured by the turbine and the available power in the wind due to the flow. The mathematical representation of the power coefficient is expressed in Equation (2) [28,32].

(2) $$C_{p}=\frac{Mechanical\;Power\;\left(P\right)}{Wind\;Power\;\left(P_{w}\right)}=\frac{P}{\frac{1}{2}\rho A{v_{w}}^{3}}$$

where, *P_w_* is the power of wind flowing at *v_w_* and can be expressed by *P_w_* = ½*ρAv_w_*^3^.

Now, the mechanical power (*P* = *τ* · *ω*) captured by a rotating object is defined as a product of torque (*τ*) and angular velocity (*ω*). Here, the *C_p_* can also be expressed as Equation (3).

(3) $$C_{p}=\frac{\left(\tau \cdot \omega \right)}{\frac{1}{2}\rho A{v_{w}}^{3}}$$

Torque applied by wind is a function of density (*ρ*) and velocity (*ν_w_*) of wind, area of turbine rotor (*A*) and diameter of transmission gears (*d*), and can be expressed by Equation (4). Therefore, the power coefficient (*C_p_*) again can be given by Equation (5) [28].

(4) $$\tau =\frac{1}{2}\rho A{v_{w}}^{2}d$$

(5) $$\;C_{p}=\frac{\frac{1}{2}\rho A{v_{w}}^{2}d\cdot \omega }{\frac{1}{2}\rho A{v_{w}}^{3}}=\frac{1}{v_{w}}\left(d\omega \right)$$

**Figure 2:**
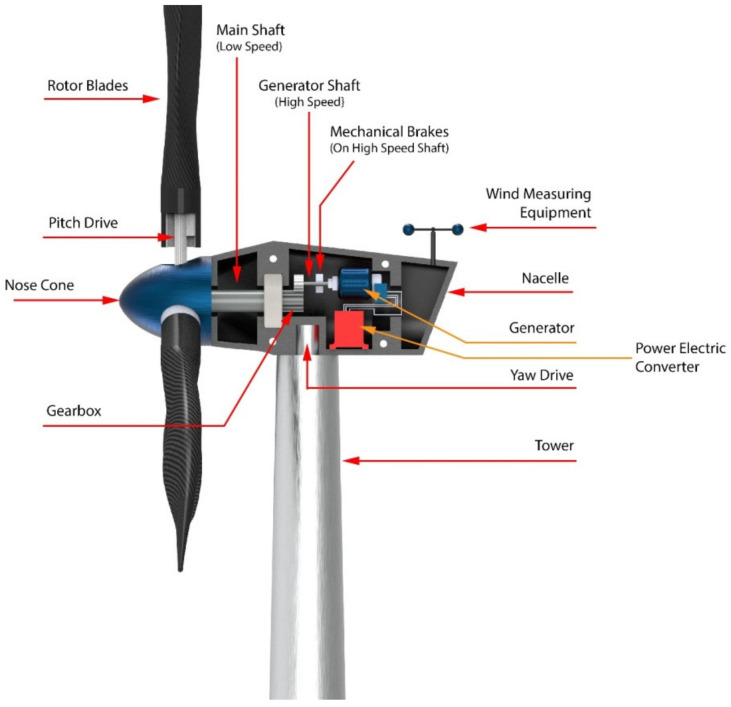
Components of WECS.

The Equation (5) demonstrates that the value of *C_p_* is directly proportional to the *ω* and inversely to the velocity of wind stream (*ν_w_*). This implies that at a constant wind stream velocity, higher turbine speed yields higher coefficient of power. This equation demonstrates that with increasing wind velocity the coefficient of performance decreases. Since power generated by the turbine is a function of *C_p_*, the power produced decreases as well. Thus, the drivetrain experienced an increased mechanical stress, while the power conversion efficiency falls significantly, nullifying the use of fixed speed turbines in commercial purposes.

b. Variable Speed WECS: The variable speed WECS were innovated from the older designs, that is the fixed speed WECS, considering the limitations of the latter, as utilization of wind power in power systems started to grow. Components of WECS are depicted in Figure 2 [33]. The limitations of the older designs were pronounced in places where the grids were not as strong to compensate for loss of power at higher speeds of wind. The development of power electronic convertors paved the way for large scale applications of the variable speed WECS. Power electronics are employed to make a connection between the turbines and the grid. The main advantage obtained by this system was that the rotor speed can be controlled according to the speed of wind. Thus, this system is able to maintain the optimum tip speed ratio by producing even and smooth power throughout the operational range. Since the optimum ‘*λ*’ is maintained, the system can thus maintain the designed value of *C_p_*, which indicates maximum aerodynamic efficiency. It is a relatively complex and costly system, however the high yield of the system compensates for these shortcomings. Present wind energy systems use them on a large scale.

## 3. Components of WECS

The WECS is an integrated system comprised of a symphony between the mechanical and electrical components. Among the various designs of wind energy conversion systems previously discussed, some components bind them together. These systems indeed share lots of components, which are able to convert the kinetic energy of wind into electrical energy in order for it to be stored in the cells efficiently. One of the biggest parts of grid connected WECS is the wind turbine assembly itself, which is the biggest tangible component. The wind turbines are compound mechanical machines built up of several small components responsible for feeding the electrical components. The aforementioned Figure 1 and Figure 2 represent the cross section of a typical horizontal axis wind turbine. A typical classification of the components can be: mechanical components, electrical components, and control system components.

### 3.1. Mechanical Components

Mechanical parts of a wind turbine have a great role in conversion of the energy. The flow of wind has some energy in it due to their kinetic nature. The mechanical regime caters to introduce a ‘work’ in the system by allowing a mass to rotate and transmit it to the desired location to interact with the electrical components. The rotor subassembly is the rotating body, which includes rotor blades, rotor bearings, nose cone, hubs, and the pitch drive system.

**Figure 3:**
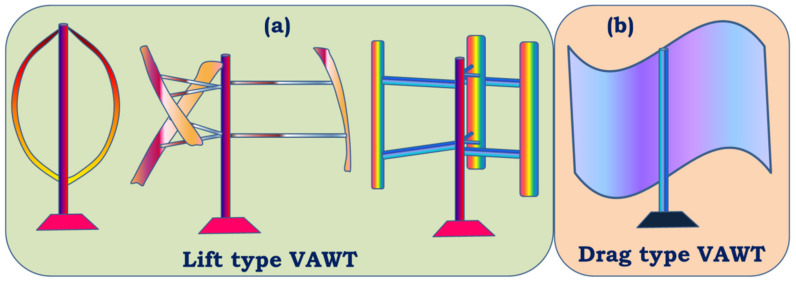
(**a**) Lift type vertical axis wind turbines (VAWTs) and (**b**) Drag type VAWT.

Some of the major mechanical components that are characteristics of WECS are:

- Rotor: Rotor assembly is the aggregate of the barely distinctive elements that interact with the wind. The assembly consists of the blades, the hub and the nose cone. The blades are the aerodynamic devices responsible to initiate the drive. In HAWTs, they resemble the propeller of aeroplane, which operates on similar principle. The blade of a typical horizontal axis wind turbine generates lift, which turns the blade during wind flow in the axial direction. The same is not true for a vertical axis wind turbine, which can either be a lift type (which uses the lift forces generated by the moving air to produce the rotation of turbine blades, see Figure 3a) or a drag type (which uses the drag force of wind to produce the rotation motion of blades, see Figure 3b). A lift type vertical axis wind (VAW) is capable of having a higher aerodynamic coefficient, and thus are used more, however, they are mostly incapable of self-start since the torque produced is lower. Over the years, research is being conducted to find the ideal materials for production of the rotor blades. Initially, they were made from heavier metals, which were resistant to environmental conditions such as high turbulent wind, however were rendered not helpful since metals tend to have higher density and thus higher mass. Concentration of mass away from the center catered to a higher rotational moment of inertia, thus diminishing the acceleration of the rotor. With a higher mass concentration away from the center, the moment of inertia of the system gets high. As a result, the acceleration gets lower, which in turn results in less response to wind speed. A massive component would only begin to be affected by winds that can exert a minimum value of force, effectively increasing the cut-in speed. In addition, another downside would be the cut-out scenario, which would require more energy dissipation through braking to keep the rotation below a certain speed, in case of high wind speeds. This turned the focus towards composites that provide rigidity while not contributing to mass. The hub of the rotor facilitates mechanical support for the rotor assembly to stay upright while the latter is rotated. It houses the bearing to provide relative motion between the fixed support and the shaft. The nose cone is integrated with the hub, which primarily directs the wind interacting with the center of the rotor towards any of the three blades. Traditional rotor designs mandated a rigid connection between the hub and the blades. The modern designs have deviated from it to include a pitch control mechanism responsible for rotating the blades about its own axis to reduce lift and control the speed of the turbine [34].
- The Main Shaft: The main shaft is a solid extrude made from any of forged high carbon steel or cast iron/steel that attaches the gearbox to the rotor hub. At present, the megawatt size wind turbines rotate very slowly and the torque is high. Thus, the main shaft is also called low-speed shaft. For example in a 5 MW power turbine, the input shaft speed is about 12 rpm while the torque is in the range of 4000 kNm [35]. This torque value can be considered to be high from a structural point of view, as a torque transmission of 4000 kNm would require approximately a 9 m cross section of EN316 type steel.
- Gearbox: The main shaft rotates slowly at a very high torque. These rotational parameters are unusable by the generator, which require high speed low torque input. The gearbox is thus included between the main shaft and generator. Modern megawatt wind turbines operate on an overall torque reduction of 60 to 120 times [36]. Research, by Nejad et al., demonstrated the use of a gearbox with an overall gear ratio of 1:96.354 [35]. For such high reduction, multi-stage gearboxes are important where one or more stage incorporates a planetary gear system, since they are capable of huge gear ratios in a small assembly space. Since gearboxes are subjected to varying torques as the input shaft of the gearbox is bolted directly to the rotor hub, the gear train experiences high wear and tear.
- Mechanical Brakes: Brakes are safety equipment installed inside the housing for emergency situations for stopping the turbine during storms or high velocity winds. They are installed directly on to the low torque high-speed generator shaft for efficient braking, rather than on the high torque low speed main shaft. This keeps them from overheating and failing during emergencies by keeping the braking torque low. Modern high-power turbines are fitted with hydraulic or electromechanical actuated brakes (both disc and drum type brake mechanisms). Braking from maximum speed of the generator shaft can cause high wear of the latter and can also cause fire hazards due to increased temperatures. Hence, they are used after proper actuation of pitch and/or yaw drives, which reduce the initial speed of the rotor.
- Nacelle: It is the enclosure housing most of the mechanical components. In horizontal axis wind turbines, the nacelle is the structure mounted on the tower behind the rotor. The size and shape of the nacelle is influenced greatly by the design of the gearbox and reduction required. This fiberglass construction is constructed to reduce turbulence to enable less vibration through the structure. Manolesos et al. demonstrated the aerodynamic properties of generic wind turbine nacelle designs [37]. Vertical axis wind turbines feature transmission and gearbox assembly at the base of the tower, thus, neither an aerodynamic enclosure nor light weight construction is necessary.
- Pitch and Yaw Drives: Pitch of turbine blades refer to the rotational degree of freedom about the longitudinal axis of the blade and the yaw is the same for the top of the turbine (including the rotor and nacelle) with respect to the tower. With regard to pitch in the classification of WECS, as we have discussed, every turbine is rotated to function at a certain wind speed and produce a designed power. Since the power generation of a turbine depends on the energy captured from wind during higher velocity of operational winds, the blades are “pitched” to alter the value of aerodynamic efficiency *C_p_*. Each blade is fitted with independent pitch control mechanism for this purpose. Likewise, the yaw drive is employed to align the turbine according to the direction of wind flow in order to obtain maximum efficiency. Discussion about control mechanisms and strategies would be covered further with greater details.
- Wind Measuring Equipment: As mentioned in regard to the pitch and yaw drives, the turbines operate on complex mechanisms, however the control of those mechanisms is ultimately managed by monitoring environmental conditions. Thus, turbines are further equipped with any kind of mechanical anemometer to measure the wind velocity around the system. An anemometer is a miniscule vertical axis turbine like arrangement with cupped struts, mounted on to the nacelle. Further, wind vanes are utilized in some wind turbines for judging the direction of flow, based on which of the controller signals the yaw drives to align itself to the wind.

### 3.2. Electrical Components

**Figure 4:**
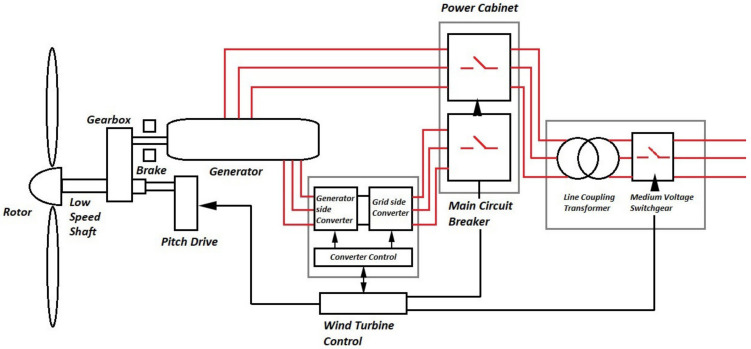
Block Diagram of a typical grid-connected WECS.

Electrical components are separated into two segments, some of which are located inside the nacelle alongside the mechanical components, while others are placed on the ground away from the tower. The chief function of the electrical subsystem is to convert the mechanical energy gathered from the wind as torque and speed into electrical energy. A block diagram of a typical grid-connected WECS is depicted in Figure 4 [28]. The step by step conversion begins with the generator, power electronic converters, transformer, wind farm collection point, and power channels etc.

- Generator: A wind generator is an electromechanical component responsible for converting rotational motion into electrical power, thus converting rotational kinetic energy into electric potential. A typical AC generator works by moving a conductor loop in a static magnetic field based on the electromagnetic induction law of Faraday. Traditionally various types of generator have been in use with the introduction of a squirrel cage induction generator (SCIG) almost 35 years ago [28,36] along with a wound rotor induction generator (WRIG). Other generators being used are doubly fed induction generators (DFIG), permanent magnet synchronous generator (PMSG), and wound rotor synchronous generator (WRSG). The DFIG based WECSs have the stator present in the generator tied to the grid, while the rotor is linked to power converters [7]. Ideally, generators are optimized to produce power at the rated wind speed, above which control systems start operating to protect the systems from damage and brakes are applied on to the generator shaft [36]. Popular choices of generator amongst WECS are DC, AC Synchronous and AC Induction [36].
- Power Converter: The output electric parameters from the generator vary widely according to the wind speed. Thus, each turbine would produce different voltages and frequencies of current at a particular instant, which is not possible to be directly coupled to the grid without refining. A power converter thus acts as an interface for connecting the turbine to the grid. The power converter transforms the output voltage from AC to DC using a rectifier and again the rectified DC to AC, having a constant voltage and frequency in subsequent steps using inverter circuit [36,38].
- Step-up Transformer: The output from the generator in a grid connected MW turbine is rather low, in the range of 400 to 690 volts, which requires it to be stepped up since the grid connects directly to the high-tension lines [39]. The necessity of a step-up transformer can be annihilated by designing the generator and power electronic converter according to the wind farm collection point voltage, however it demands additional costs for medium voltage generator and power converter setup, diminishing the economic benefits of removing a step-up transformer from the circuit [40]. However, studies are being conducted in recent years to eliminate the needs of transformers in medium voltage grids in order to reduce transmission losses incurred from stepping up the voltages required for transmission [39,41].
- Wind Farm Collection Points or Point of Common Coupling: The point of common coupling (PCC) is a connecting ground of all the turbines of a wind farm. The preferred mode of connection in the wind farms is a parallel connection, which facilitates either maintaining a desired potential difference or having a defined node for connecting more turbines if necessary [41].

## 4. Flow of Power in a WECS

The WECS is a constant speed or constant frequency device, which is fitted with an induction generator, which is operated by a wind turbine without any controller. The power content in a stream of wind is a function of wind flow velocity. When the speed of wind stream is *V*, the power (*P*), which is a rate of change of kinetic energy of wind according to definition can be expressed in Equation (6) [42,43].

(6) $$P=\frac{dE}{dt}$$

Kinetic energy of a system is given as product of mass (*m*) and square of velocity (*V*) as given in Equation (7) [12,13].

(7) $$E=\frac{1}{2}mV^{2}$$

Inserting the expression of kinetic energy in the Equation (6), we get, $P=\frac{d}{dt}\left(\frac{1}{2}mV^{2}\right)$. Differentiating the expression with respect to *t*, $P=\frac{1}{2}\left(V^{2}\frac{dm}{dt}+2mV\frac{dV}{dt}\right)$. Since the wind velocity, *V* is constant, thus, $\frac{dV}{dt}=0$. Therefore, the relation of power with mass flow rate of the wind ($\dot{m}$) is arrived at from energy in Equation (8) [44].

(8) $$P=\frac{1}{2}\dot{m}V^{2}$$

Let the cross-sectional area of the wind column in consideration be *A* and the air density be *ρ*. Therefore, the mass flow rate can be written below in Equation (9) [43].

(9) $$\dot{m}=\rho AV$$

Therefore, Equation (8) can be rewritten utilizing the value of mass flow rate from Equation (9). This can be written as given in Equation (10) [44].

(10) $$P=\frac{1}{2}\rho AV^{3}$$

The Equation (10) represents the power content in a steam of wind only due to its flow and velocity. However, similar to Carnot heat engine, a turbine system cannot theoretically extract the whole power from the flow. This is governed by Betz Equation, which deals with upstream velocity of wind, *V*_1_ and downstream velocity of wind, *V*_2_ and the maximum extractible power is called the Betz limit. The wind turbine cannot extract the whole available power due to breaking of wind velocity from *V*_1_ to *V*_2_, allowing a continuation of wind regime [44]. The estimation of Betz limit assumes the turbine to be ideal, that is:

- The turbine has no hub and has an infinite number of blades with no drag from any of the blades (ideally zero drag);
- The flow is non-compressible, that is, the density of the air remains constant throughout;
- Thrust experienced by the rotor is uniform throughout.

Betz equation further considers the flow to be axial to the turbine. The ideal turbine is in a non-rotational form at the time of placing it in the wind atmosphere. The cross-sectional area of swept by the blade is A while the cross-sectional area upstream and downstream are designated the values S_1_ and S_2_, respectively.

The speed of wind interacting with the rotor is taken to be *V* while the velocity of wind upwind from the rotor is designated to be *V*_1_ and the downstream velocity is designated to be *V*_2_. By definition, the turbine extracts mechanical energy from the wind by reducing its kinetic energy from the upstream to the downstream. Therefore, *V*_1_ would be greater than *V*_2_ and subsequently, the cross-sectional area enhances from the upstream to the downstream, and hence, S_1_ would be lesser than S_2_. Since the stream of air is considered incompressible from the listed assumptions, the mass flow rate remains constant throughout.

The force experienced by the rotor of the wind (*F*) can be obtained by applying Euler’s theorem [45] i.e.,

$$F=ma=m\frac{dV}{dt}=\dot{m}\Delta V$$

Power flux is an essential fluid parameter, which defines the rate of energy flowing through a given cross-sectional area. By definition, power flux (*P′* = *P/A*) is the rate of energy flow per unit area. The cross-sectional area is pre-defined to be *A*, and the rate of energy flow is effectively the power flow, *P*.

Using general value of *P* from Equation (9), we can arrive at the relation given by Equation (11).

(11) $$P^{\prime }=\frac{\frac{1}{2}\rho AV^{3}}{A}=\frac{1}{2}\rho V^{3}$$

The kinetic power content in the air upstream to the rotor is obtained by replacing the velocity term with upstream velocity *V*_1_ in over a cross-sectional area *A*, which is demonstrated in Equation (12).

(12) $$W=\frac{1}{2}\rho A{V_{1}}^{3}$$

We can introduce a dimensionless number obtained as the ratio of the net power extractable from wind, to the total power content of the same wind stream. This designates the coefficient of performance or efficiency of extracting power. The coefficient of performance represented by *C_p_* is also known as the aerodynamic efficiency since turbines rotate by the action of aerodynamic properties of the blades. It signifies the available power percentage extracted by a turbine. The *C_p_* value can be expressed in terms of *P* and *W* and we get a simplified relation as expressed in Equation (13) [42,44].

(13) $$C_{p}=\frac{P}{W}=\frac{\frac{1}{4}\rho A{V_{1}}^{3}\left(1+b\right)\left(1-b^{2}\right)}{\frac{1}{2}\rho A{V_{1}}^{3}}=\frac{1}{2}\left(1+b\right)\left(1-b^{2}\right)$$

Equation (13) gives a sensible relation between the coefficient performance and velocities of wind before and after hitting the turbine. Setting the value of *b* as zero in Equation (13), we get *C_p_* to be 0.5 and setting *b* as 1, we get *C_p_* to be zero. Thus, the nature of the curve needs to be investigated in this domain. Moreover, putting *b* = 1 gives *V*_1_ = *V*_2_, which represents no kinetic energy being extracted from the wind and *b* = 0 gives *V*_2_ = 0, which represents no wind flow beyond the turbine. Between the points *b* = 0 and *b* = 1, a point of zero slope would give the maximum value of *C_p_*. Therefore, differentiating *C_p_* with respect to *b* when$\;\frac{dC_{p}}{db}=0$, will get a relation as derived in Equation (14) [46].

(14) $$\frac{dC_{p}}{db}=\frac{1}{2}\frac{d}{db}\left[\left(1+b\right)\left(1-b^{2}\right)\right]\;\;or,\;\;\;0=\frac{1}{2}\left(1-3b\right)\left(1+b\right)$$

The Equation (14) is derived to find a suitable solution. The equation holds for two values of *b* = −1 or $b=\frac{1}{3}$. When *b* = −1, $\frac{V_{2}}{V_{1}}=-1$ or *V*_2_ = *V*_1_.

This gives a trivial solution for the Equation (11) since the direction of the wind cannot be reversed and the range of *V*_2_ is in positive values of *V*_1_. Therefore, this solution should be ignored when $b=\frac{1}{3}$, $V_{2}=\frac{1}{3}V_{1}$. Thus, the velocity is decreased by two-thirds and the initial stream velocity for maximum achievable *C_p_*. This has been validated experimentally in a wind tunnel and mathematically modeled in line with many other studies [18]. Therefore, the value of $b=\frac{1}{3}$ can be used in Equation (13) to find the value of *C_p_*, which is expressed in Equation (15) [46].

(15) $$C_{p}=\frac{1}{2}\left(1+\frac{1}{3}\right)\left[1-{\left(\frac{1}{3}\right)}^{2}\right]≅0.59\;or,C_{p}=0.59$$

This value of $C_{p}$, obtained in Equation (15), indicates that with highest possible design optimization, the efficiency is still 59% of the available wind energy. Therefore, the Betz coefficient value (i.e., 16/27 ≈ 0.593) indicates that a wind turbine can extract, at most, 59.3% of the energy in an undisturbed wind stream [44]. It is thus the target of modern manufacturers to extract 100% of this extractable value. Recent advancements in technologies have been able to push this value to about 75% to 80%, which is around 45% to 48% of the available wind energy in the free stream [44,46,47].

## 5. Control Strategies Employed in WECS

As discussed earlier in Section 2.3, the control systems are useful for a turbine for its safe operation. However, to drive those systems, strategies are utilized so that the turbine can generate optimal power for various atmospheric conditions while increasing the life span of the structure by controlling the loading conditions. The strategies determine the operational parameters and change the configuration such as pitch angle, yaw angle, etc. The grid connected WECS incorporates some of the control strategies that will be discussed in this paper. The controls associated to modern day wind energy conversion systems are broken down into six levels of controls, which are chronologically numbered. The first level, or the Control Level 1, deals with slow changing parameters while the Control Level 6 handles the fast-changing parameters. The Level 1 is responsible for imposing power commands to other control levels. The loops in the control hierarchy also monitor abrupt functions of the WECS.

Control in Level 1: Transmission system and distribution system operator (TSO/DSO) controls
Control in Level 2: Wind farm centralized control
Control in Level 3: Wind turbine centralized control

This level can be broken down into mechanical and electrical controls.

Mechanical Controls:

- Pitch control
- Yaw control

Electrical Control:

- Reactive power generation (RPG)
- Fault ride-through (FRT)

Electro-Mechanical Controls:

- Damping control
- Ancillary services

Control in Level 4: Grid connection (GC) and maximum power point tracking (MPPT) control

- Integration and synchronization of grid
- MPPT control

Controls in Level 5: Generator and control of grid

- Speed, torque and power control of generator
- Voltage oriented control of grid

Controls in Level 6: Power control converter

- Current control of generator
- DC chopper control
- Current control of grid

The whole operation of control works on a system of feedback. In case of faults in the grid, the fault ride-through control of control level 3 generates a fault enable signal, signifying a positive identification for a fault, which is responded to by coordinating for better control performance by mechanical and electrical controls included in control levels 1 to 6. Feedback signals in a traditional wind energy conversion system consist of voltage and current of grid, angular speed along with voltage and current of generator, DC link voltage conditioner, a rotor positioning with respect to mean position and wind speed from the anemometer mounted on the nacelle [48].

Level 1: Transmission system operators or distribution system operators supervisory control.

The output from the turbine or an entire wind farm is not uniform; rather it varies with undulations in wind, which cannot be controlled. The coupling of this unstable energy waveform into the existing pool of energy causes reliability issues and the excessive penetration of a large scale WECS into the existing power system. The WECSs are linked to the TSO/DSO dispatch centers and keeps them updated about the active as well as reactive power generation statuses on a continuous basis. The TSO/DSO sends active and reactive signals based on the received status signals to the respective wind farm setup. Over the years, the large-scale wind farms have changed their operation from being passive sources of power to active ones through constant efforts made by the TSO/DSOs.

Level 2: Wind farm centralized control.

This acts as a communicative medium between the wind farm and the level 6 control. Every turbine in a wind farm is connected to the centralized hub through communication lines. Through it, the status of the WECS is shared to the TSOs and DSOs. The data acquisition and supervisory control systems are responsible for monitoring the operation of the wind farms. The active and reactive power requirements are defined for each specific wind turbine in a farm by the centralized control of that farm, which are transmitted chronologically as per the number of turbines it is in the farm. Since the control is highly reliant on feedback channels, the static compensators of the wind farms, including static synchronous compensator (STATCOM) and static var compensator (SVC), are initiated to meet the reactive power generation (RPG), when the centralized control realizes it is unable to do so normally. Besides all the operations required to meet the references imposed by the higher level, i.e., level 6, the centralized control also checks the aerodynamic interactions.

Level 3: Wind Turbine Centralized Control.

The level 4 of the control system is a cluster of electrical, mechanical and electro-mechanical systems as we have established in the classification. The pitch and yaw control mechanisms are responsible for changing the physical operating parameters and come in mechanical controls, whereas RPG and FRT are responsible for signal modifications, establishing the electrical controls. The third control includes damping, which can be both mechanical as well as electrical. Mechanical damping is necessary to protect mechanical components from torsional vibrations in the drivetrain and resonances in the tower caused by wind, whereas electrical damping provides protection to electrical counterparts by damping electrical sub-synchronous resonance in grids.

The ancillary service acts as an electrical back-up for the mechanical control system and includes the power storage module as well as an uninterrupted power supply (UPS). The energy storage module collects power from idle spinning of the rotors without wind, i.e., rotation due to inertia after wind has fallen below cut-in velocity [49]. As mentioned previously, the MW—powered wind turbines equipped with pitch control mechanism can change the rotation of the blades about their vertical axis. A proportional integral (PI) controller is dominantly uses a thyristor and rectifier kept in between the generator and resistive load. The PI controller works by calculating the integral error to the reference maximum power [32,48]. The PI calculates the exact value of the pitch angle, when the wind speed exceeds the rated wind speed, so that the output power from the generator is limited to its rated output value. However, at wind speeds below the rated one, the pitch angle is kept at zero for maximum possible energy extraction [32,48].

Level 4: Grid connection and MPPT control.

MPPT signifies the control loop that is associated with extracting the peak power at all times. The loop works by using a suitable algorithm on control to adjust the phase between the grid current and grid voltage for it to correspond with the grid power factor requirement. Synchronization of grid parameters is possible by different methods such as zero-crossing, grid voltage filtering and phase-locked loop. Among them, the phase-locked loop is preferred due to the robustness of the controller against the voltage harmonics and simplicity of the platform [49].

With the levels defined, we can now move on to discuss the strategies undertaken by the WECS. Depending on the type of generator used, the control strategies can be broken down as the generator based WECS. The DFIG exhibits the salient feature of variable operational speed due to the integration of the variable resistor in the circuit. Presently, the widest used generator in WECS is DFIGs. The following controls are therefore in the context of DFIG based WECS.

- Optimal TSR (OTSR) Control: The maximum peak point tracking as discussed previously, utilizes algorithms to estimate reference operational speed ($\omega_{m}^{*}$) for the prevalent wind speed ($v_{w}$) considering other physical parameters of the turbine such as pitch angle, as well as feedback signals for required power factor. The $\omega_{m}^{*}$ is tuned based on the wind velocity, such that the optimum tip speed ratio ($\lambda_{T}^{op}$) can be churned. The adjustment of the speed is done based on the expression as given in Equation (16):
  (16) $$\omega_{m}^{*}=\omega_{T}^{*}r_{gb}=\left(\frac{\lambda_{T}^{op}r_{gb}}{r_{T}}\right)v_{w}$$
  where, $\omega_{T}^{*}$ is the angular velocity of the rotor; $r_{gb}$ is the gear ratio employed in the gearbox and $r_{T}$ is the radius of the rotor, in compliance with Equation (1). The control levels 1 and 2 operate on feedback from the generator. The feedback from generator is a function of the rotational speed of the generator. The control level checks for the error between the obtained value and the reference velocity for every instant and attempts to minimize this error. As this control technique does not involve memory, since the data are processed and compared in every instant, the complexity of the system is lowered. This MPPT control strategy is widely in use in WECS industry, being the simplest among all MPPT control strategies [50,51]. However, proper measurement of wind speed is an important criterion, which demands the use of expensive equipment. Ultrasonic sensors are promising in this regard due to their ability to furnish reliable and accurate wind information.
- Turbine Power Curve (TPC) based Control: Similar to the previous control method, MPPT strategy utilizes the wind speed for controlling the turbine speed. In this process, the power produced based on wind speed curve is referenced to the power curve provided by the manufacturer of the wind turbine, which is initially tested in controlled wind speeds, which provides the output power curve. Each wind speed and corresponding power, $P_{0}^{*}$ output is stored in a memory space. During operation at a particular wind speed, the power produced by the generator can be compared with the data point at a same wind speed from memory. Here, the output signal from the generator is in terms of instantaneous voltage, $V_{0}$ and current, $I_{0}$. Power is obtained as $P_{0}=V_{0}\cdot I_{0}$. The control levels 1 and 2 attempt to reduce the error between the power values $P_{0}^{*}$ and $P_{0}$ by response signals, which closely resemble the ones used in “optimal tip speed ratio control” [52].
- Optimal Torque (OT) Control: The previous methods rely completely on the speed of wind to govern the control of the operational parameters. Therefore, even with the implementation of expensive wind speed measurement equipment, the input information is still incomplete. Thus, for increased reliability, other MPPT control techniques also utilize the rotational speed of the generator rotor. In this, the speed of the generator is used to calculate the reference electromagnetic torque $T_{e}^{*}$.

We can conveniently rearrange the expression in Equation (2) to get the power produced by the rotor and by substituting the value of $v_{w}$ into this equation from Equation (1), we get the mechanical power as Equation (17):

(17) $$P=\frac{1}{2}\rho A{v_{w}}^{3}C_{p}=\frac{1}{2}\rho A\frac{{\omega_{T}}^{3}{r_{T}}^{3}}{{\lambda_{T}}^{3}}C_{p}$$

The turbine torque (*T_T_*) and the turbine speed (*ω_T_*) can be stated by Equation (18) and Equation (19), respectively:

(18) $$T_{T}=\frac{P_{T}}{\omega_{T}}$$

(19) $$\omega_{T}=\frac{\omega_{m}}{r_{gb}}$$

where, *P_T_* = Power output from turbine, *ω_T_* = rotational speed of turbine, *ω_m_* = rotational speed of generator, *r_gb_* = gear ratio of gearbox.

The torque experienced by the generator from the drivetrain is defined by $T_{m}=\frac{T_{T}}{r_{gb}}\;$. At steady state conditions, the electromagnetic torque (*T_e_*) produced by the generator should be equal to the mechanical torque (*T_m_*) experienced from the drivetrain. Therefore, at *T_e_* = *T_m_*, from the above relations, the torque can be written as a function of angular velocity of the generator by Equation (20):

(20) $$T_{e}=T_{m}=\frac{T_{T}}{r_{gb}}=\frac{P_{T}}{\omega_{T}r_{gb}}=\left(\frac{1}{2}\rho A{r_{T}}^{3}\frac{C_{p}}{{\lambda_{T}}^{3}\cdot {r_{gb}}^{3}}\right){\omega_{m}}^{2}$$

The reference electromagnetic torque considers optimal coefficient of power and tip speed ratio. Therefore, the reference torque is given by Equation (21):

(21) $$T_{e}^{*}=\left(\frac{1}{2}\rho A{r_{T}}^{3}\frac{C_{p}^{op}}{{\lambda_{T}^{op}}^{3}{r_{gb}}^{3}}\right){\omega_{m}}^{2}=K_{op}{\omega_{m}}^{2}$$

In Equation (21), *K_op_* can be treated as a constant for the operation and can be calculated initially with the known design parameters. The torque can be obtained by multiplying this with the angular velocity of the generator. The electromagnetic torque follows a quadratic relation with the speed of the generator evident from the expression. The control in this strategy employs control levels 1 and 2 to equalize the electromagnetic torque to the reference value, by referencing during operation. This method is also used on a large scale in commercial MW-WECS [52].

d. Power Signal Feedback (PSF) Control: Another MPPT control utilizes the input signal in the form of generator speed. This follows the basic principles of Power Curve based control strategy, however instead of monitoring the wind speed, sensors are employed to monitor the speed of the generator. The Equation (16) signifies that the *ω_m_* of a generator is directly proportional to the *v****_w_***. Therefore, similar to the power curve used in the wind turbine power curve–based control, this strategy utilizes power versus wind velocity curve to compute the reference power, $P_{0}^{*}$. The control system utilizes a fuzzy logic–based controller that has two inputs, namely, the rotor speed from the generator and the generated power. The estimated output power can be generated corresponding to the conditions. If the input power to the controller is lower than the estimated maximum value, the generator is controlled accordingly to reach more velocity allowing more power to be elicited from the wind [53].
e. Speed Sensor-less (SSL) Control: This MPPT control operates without the use of any kind of speed sensing device. Rather, the wind speed is predicted by autoregressive statistical models of WECSs, which are based on the measured frequency (*f_a_*) of the generator. The predictions implemented regarding the wind speed category based on the basis of time scales are [54]:
  - *Ultra-short-term forecast*: These predictions are regarded for wind conditions about an hour ahead.
  - *Short-term forecast*: This is applicable for forecasts several hours ahead.
  - *Medium-term forecast*: The prediction of wind conditions in the time span from a few hours to about a week.
  - *Long-term forecast*: Used for detecting wind conditions more than a week ahead.

**Figure 5:**
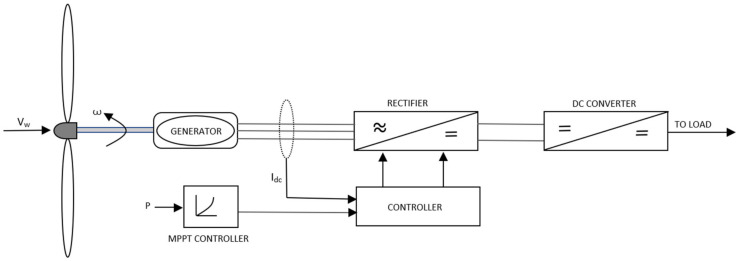
Schematic of Hill Climb Search Control.

**Figure 6:**
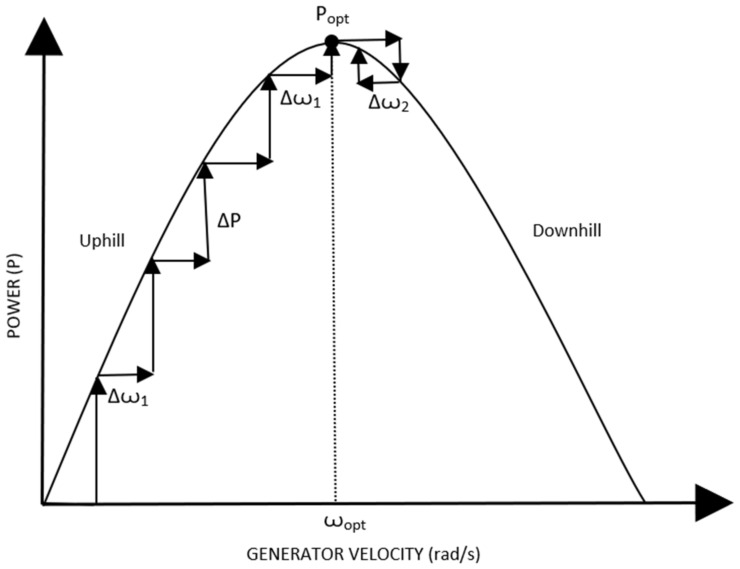
Power curve in a HCS Control.

The predicted wind speed is used to calculate the reference power, $P_{0}^{*}$ similar to the process incorporated in power curve-based control. Since the wind speed is predicted in this format, the method can also be used congruent to optimal tip speed ratio control, however the increased complexity of this control system impedes its use on a large scale. A summary of MPPT control techniques used for DFIG based WECS is illustrated in Table 1 [28,53].

f. Hill Climb Search (HCS) Control: It is a method of maximum peak point tracking control strategy that involves a disturbance in the reference signal. The inputs in this control are obtained in the form of generator speed and dc-link current. The output is a command signal that is needed for extraction of maximum/peak power. A schematic of the hill climb search control is depicted in Figure 5 [53]. As the generator speed increases, the output power is seen to increase while approaching the peak output power, as illustrated in Figure 6 [53]. After the peak power is encountered, an increase in rotor speed tends to decrease the power output. The mechanical power being generated is compared to the maximum extractable power in this method, after which it is decided whether or not it is necessary to increase the generator speed. The speed of the generator is altered by changing the dc-link’s current value. Once the WECS control starts, the controller perturbs for every instant by tracking maximum points with every instance of the operating parameters being changed.

The control methods discussed above are used precisely in a doubly fed induction generator (DFIG) based WECS. Other examples of WECS architecture, such as the permanent magnet synchronous generator (PMSG) and the squirrel cage induction generator (SCIG) based designs, are substantially more expensive than the DFIG. Moreover, the inverter output filters and electromagnetic interference (EMI) filters used in the previous types of WECS are rated for 1 p.u. of output power, which also contributes to the complexity of designing the filter, making it more costly. The WECS employing DFIG at its core uses back to back converters. The primary advantage thus obtained is the reduced converter rating, which is about 30% of the generator rating; while the speed range of variable speed WECS stays at about 33% of the synchronous speed mechanisms [55].

A PMSG is a staple in the industry for innovating and incorporating new designs, and has attributed to it a higher efficiency alongside greater power density and an achievable smaller diameter of turbine in direct-drive systems. These are used particularly in older generation turbines to keep the complexity lower. Permanent magnet materials of high energy are also available at quite a reasonable price, making these generators desirable. With increasing wind farms, the focus is concentrated on achieving WECS which is reliable, and has lower maintenance cost and noise while being efficient. This converged to direct drive PSMG based WECS. A conclusive design consisting of three MPPT based controls is applied on available wind turbines.

**Figure 7:**
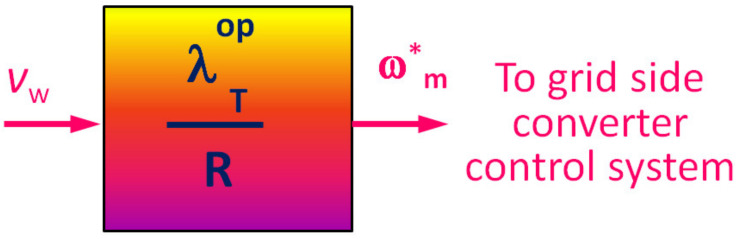
Tip Speed Ratio (TSR) Control.

- Tip Speed Ratio (TSR) Control: Not unlike the optimal TSR control strategy used in case of a DFIG based WECS, the optimal TSR (OTSR) is evaluated with the velocity of wind, *v_w_* as an input parameter. A schematic flow of TSR control is depicted in Figure 7. With the knowledge of the tip speed ratio to be maintained for the given wind speed, the optimal speed of the turbine is to be evaluated by the known relation given by Equation (22) [28,53]:
  (22) $$\omega_{m}^{*}=\frac{\lambda_{T}^{op}}{R}v_{w}$$
  where, *R* is the turbine rotor radius. This speed control command is used further to the control loop responsible for controlling the speed. The PI controller is responsible for processing the command and to limit the speed of the turbine rotor to the desired maximum/peak value of rotor speed obtained by the evaluation.
- Power Signal Feedback (PSF) Control: The power signal feedback system of control utilizes the information about the rotor speed as input of the controller and power produced as a feedback, directly from the generator. In this system of control, the reference power is evaluated through the algorithm, which is then compared to the produced value of power obtained through feedback loop.

We know that the power produced at any instance depends on torque (*T*) and angular velocity (*ω*). Therefore, using Equation (21), it is easily perceivable that to optimal power (*P*_O_) can be obtained given in Equation (23) similarly by multiplying the result with the term of angular velocity, applying minor modifications to it. Since here the rotor speed is monitored, the angular velocity of the generator has to be replaced by the angular velocity of the rotor.

(23) $$P_{O}=K_{op}{\omega_{T}}^{3}$$

**Figure 8:**
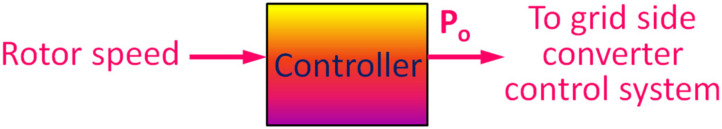
Power Signal Feedback Control Block Diagram.

Here, $K_{op}$ is a design constant, based on physical parameters of the turbine. Hence, Equation (23) gives the output as the reference power. Figure 8 illustrates this control through a block diagram [53].

Over recent years, various pieces of research have been conducted to further utilize the potential of control strategies. As a result, improvisation of already existing methods to increase effectiveness has been brought up.

Zhang et al. presented a fuzzy logic controller for the pitch angle of the turbine [56]. Traditional pitch control logics employ the use of a PI controller, as discussed in level 4 control. The implication of such controller is that the user or operator needs to have knowledge of mathematical model of the same. However, with the application of a fuzzy logic, the wind speed can be used to compensate for the non-linear sensitivity. The use of fuzzy logic controllers is apparent where the system dynamics contain multiple non-linearities like inconsistent wind speeds. The presented study incorporates triangular symmetrical membership functions for input and output parameters, as well as a mathematical model of the turbine created using MATLAB. The triangular symmetrical functions provide increased sensitivity for variable values that are approaching zero. The width of the functions can be adjusted with respect to each parameter. The outcome of the alternate fuzzy logic helped in reducing the ripples in torque as seen in conventional PID controllers, signifying a smoother torque curve.

Another MPPT control strategy was proposed by Dalala et al., in 2013, and suggested maximizing the energy captured while operating at wind speeds below the rated wind speed. This study is applicable for smaller scale WECS [57]. This strategy of control involves multiple stall controls for high wind speed regions. A constant speed stall controller allows the rotational speed of the generator to be kept limited to the rated speed, while a constant power stall controller helps to keep the generated power within permissible limits. This control strategy works on two inputs, namely, the electromechanical torque and the wind speed. To compensate for the instability of stall regions, the proposed control employs a stabilizing control loop, and a mode of transfer control strategy is innovated to stabilize the transition between various operational modes.

## 6. Present Trends in the Field of WECS and Scopes of Improvement

### 6.1. On-Going Trends

**Figure 9:**
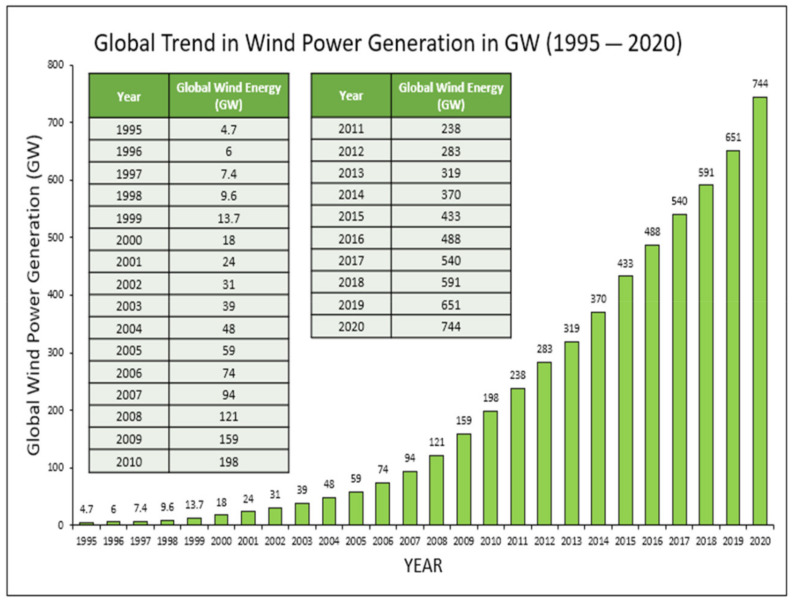
Trend in Wind Power Generation in Giga Watt (GW) [59,60].

A sharp incline in the installation of wind energy firms have been observed within recent decades throughout the world, rendering a shift of dependence on clean energy [58]. The present trend of global wind energy production has been represented in Figure 9 [59,60]. In India itself, the total wind energy capacity was found to be 21,263 MW until May 2014. With the harnessing of wind energy on such large scale, recent times have attracted research and development on every aspect of WECS.

Modern wind energy conversion systems rely almost totally on power produced by horizontal axis wind turbines. Such wind turbines are common scenarios in large commercially- and grid-connected wind farms, while the development of vertical axis counterparts have been impeded early, as they were in the later decades of 20th century. The concentration of these studies took place only in the US and UK, at the US Department of Energy Sandia Lab and Reading University, respectively [58]. However, further study on the topic ceased once it was established that VAWTs were incapable of producing power as efficiently as HAWTs could for large scale power production. The general concept of HAWT and VAWT gives way to few disadvantages for the latter. VAWTs are inefficient, as the blades move approximately half of the rotation along the direction of wind, and the other half against the same wind. This is not much of a problem for HAWTs as the part of the rotor going against the direction of wind is far below the point that traverses along the direction of the wind. Since wind at the top is stronger, due to characteristics of wind with altitude, the force driving the turbine is far higher than the force that opposes it. Another main advantage of HAWTs over VAWTs is that they can be installed at any height, however in contrast, the VAWTs must be installed close to the ground. Therefore, an installation that is not on a mast loses a great deal of efficiency in case of VAWTs due to the fact that natural wind is blown more strongly and evenly at greater heights [61].

Contrary to old research, recent developments have suggested the practicality of vertical axis turbines in the large-scale generation of power in wind firms, as they have better aerodynamic efficiency than the traditional commercial HAWTs [62]. Vertical axis wind turbines are brought back into focus for evaluation of its aerodynamic properties as well as their performances regarding flow separation. Attempts to nullify the adverse effects of energy production using these set ups are practiced [63]. Furthermore, to test the endurance and reliability of a VAWT and to meet the energy requirements, recently a VAWT has been designed according to the harsh conditions of the Amundsen–Scott South Pole Station [64], which is a scientific research station in southernmost place on the globe. Being at the extreme end of South Pole, the turbine is subjected to rather harsh weather conditions.

As stated in the previous section, the majority of research presently being conducted is to test the application of various control strategies and methodologies with different set ups. With the advancement in the past decade of software and computer technologies, the research regarding control strategies highly incorporates simulation studies. The simulations carried out for confirming the effectiveness involve a mathematical model of the turbine itself. Usage of Maximum Power Point Tracking controller for generating control strategies are focused primarily on recent studies, which is evident due to the simplicity and potential of the system [65]. As DFIG has a low inertia response, they are used in most wind turbines. The response of these DFIGs can be modified for good with the inclusion of a feedback parameter, where this would be used as a function of the rate of change of the frequency of the grid with respect to the electromagnetic torque [66]. As a response to the adaptation of DFIG for commercial purposes, it is imperative to integrate the potential of artificial intelligence (AI), for actively configuring the operational parameters. AI is being used in all aspects of society at present. Studies have shown interesting results, comparing the robustness and response time of an artificial neural network controller to a classical PI controller [67]. In every case, it has been found that the implementation of a neural network to predict the wind flow patterns have rendered more effective control. The response time could also be significantly reduced, with a higher robust operation.

Advancements in energy storage technologies are at a peak as well [64]. Even though for modern wind turbines, being directly connected to the grid does not directly require a power storage module. However, there are components that incorporate the need of batteries, such as in ancillary services that act as a backup power source for running the control systems, besides making the output to the grid far more predictable. While traditionally many devices and methodologies have been formulated to flatten the generated power curve so as to get rid of spikes in it, for the waveform received in the grid side, efficient storage technologies can also help to provide a smooth output power curve [68]. Storage technologies being investigated in recent years to find their application in wind energy systems include the following, although this list is not exhaustive [56]:

- battery;
- flow battery (FB);
- fuel cell (FC);
- flywheel (FW);
- superconducting magnetic energy storage (SMES);
- supercapacitor (SC);
- compressed air energy storage (CAES);
- pumped hydroelectric energy storage (PHES);
- water storage.

In contrast to flywheels that store energy by virtue of mechanics [69], FC and battery can store energy in the form of chemical energy, making them more practical in the intended applications. Promising technologies of battery for the use in grid-energy systems include advanced lead–acid (Pb–acid), nickel–cadmium (Ni–Cd), lithium-ion (Li+), sodium–sulphur (Na–S), nickel metal hydride (NiH) and flow batteries [63].

- Advanced lead-acid battery: Regular lead–acid batteries have been a staple for a variety of applications in every corner of industry, as well as in an automotive and domestic context. Though, most prominent and specific applications include frequency regulation, bulk storage of energy in case of integration of variable renewable energy sources and distributed energy storage systems. As a result of versatile and vast usage of the Pb–acid batteries, these have been an interesting focus for further enhancements. A conventional Pb–acid battery comprises lead–dioxide (PbO_2_) as cathode and lead (Pb) as anode. During the discharge process, chemical reactions in the cell lead to the synthesis of lead sulfate, whereas, during charging, this reaction reverses and Pb gets deposited back onto the electrodes. Recent developments have demonstrated that introducing carbon into the cell can help with the prevention of detrimental crystallization during charging and discharging cycles [56]. The implication of this is an increase in the life-span of the batteries, besides an increase in life-cycle between consecutive charging.
- Sodium-sulfur battery: The core of this battery technology houses molten sodium in an ionic state, which is lined with alumina (or aluminum oxide) on all sides. This is placed inside a sodium sulfate surrounding. The advantage of sodium sulphur battery is a high energy density, making it suitable for places of application that demand short yet potent bursts of energy. These batteries are used in rather functional contexts in energy distribution grid applications, wind power integration, in ancillary services as UPS batteries and much more.
- Lithium ion battery: As the name points out, these batteries incorporate positively charged lithium ions (as metal), that flow through electrolyte and promotes flow of electrons across the connected outer circuit. From time to time, lithium ion batteries have found a place in almost all electronic applications, alongside medical uses, owing to the far superior energy density values. At present, lithium ion batteries are being tried and tested for application in automotive industries, prompting its upcoming uses in fields of renewable energy industry [56]. Current developments are focused on improving the charging and discharging cycle efficiency of these batteries.
- Flow batteries: A flow battery technology stores energy by the virtue of chemical energy in the form of two electrolyte reservoirs inside the body of the battery. The chemical energy is converted into electrical energy when one liquid is displaced slowly and steadily from one tank to another. The displacement of the liquid when reversed acts to charge the batteries. Since they are the function of the fluid motion, they are termed as flow batteries. The energy storing capacity of these batteries is solely governed by the size of the tanks and the energy density is dependent on the volume flow rate from anode to cathode. The primary advantage, for which they are considered as a potential candidate for evolving battery technologies, comes from the fact that these batteries can have very high-power ratings, and the electrolytes can be stored and replaced easily, more conveniently than typical cell electrolytes, coupled with adjustable and replaceable flow properties according to the application.

The water storage is an unused generated energy, which is trying to be converted into potential energy from the water bring pumped from a lower container to a higher one. In the near future, its performance might be compared with chemical batteries [70].

Adaption of newer technologies has always been a lengthy process. Usually, a new technology is expensive to begin with, making them undesirable at this early stage. However, with lots of scope for improvement, those who take up the idea and make it the focus of further studies and increase understanding make them cheaper and more acceptable, besides making them superior with regard to existing technologies [71]. In this context, the novel nanomaterials would play a vital role in the near future of these renewable systems [72,73]. For example, superhydrophobic coating would enhance the shelf-life of the turbine, as well as various components of the WECS [74]. The emission properties of nanolayers could be used to investigate the erosion scars of the turbines [75,76]. Rapid adaptation of new technology in the market, coupled with the replacement of older ones, results in the effective penetration of a new product of about 15% [56]. A similar phase is being observed by wind turbine technologies.

### 6.2. Future Prospects

Since wind energy is a relatively newer source of energy, most of the resources that capture the energies have focused on the development of turbines and tower designs, as well as methods to increase the efficiency of existing systems. However, possibilities for the evolution of technologies are endless. However, a future emerging technology in this field of study would consider the following criteria to be accepted [77]:

- Technologies based on supply and conversion of wind energy;
- A technical overhaul, with new fundamentals, which cannot be a result of incremental research on prevalent technologies; and
- A technology which is in its early developmental stages.

While some of the technologies enlisted below have been used in practice, they may not have necessarily been used in wind energy systems and thus have ample space for improvements to be made usable. Even if not a completely radical innovation, large scale adaptation of such technologies might be considered as an emerging technology.

#### 6.2.1. Airborne Wind Energy (AWE)

**Figure 10:**
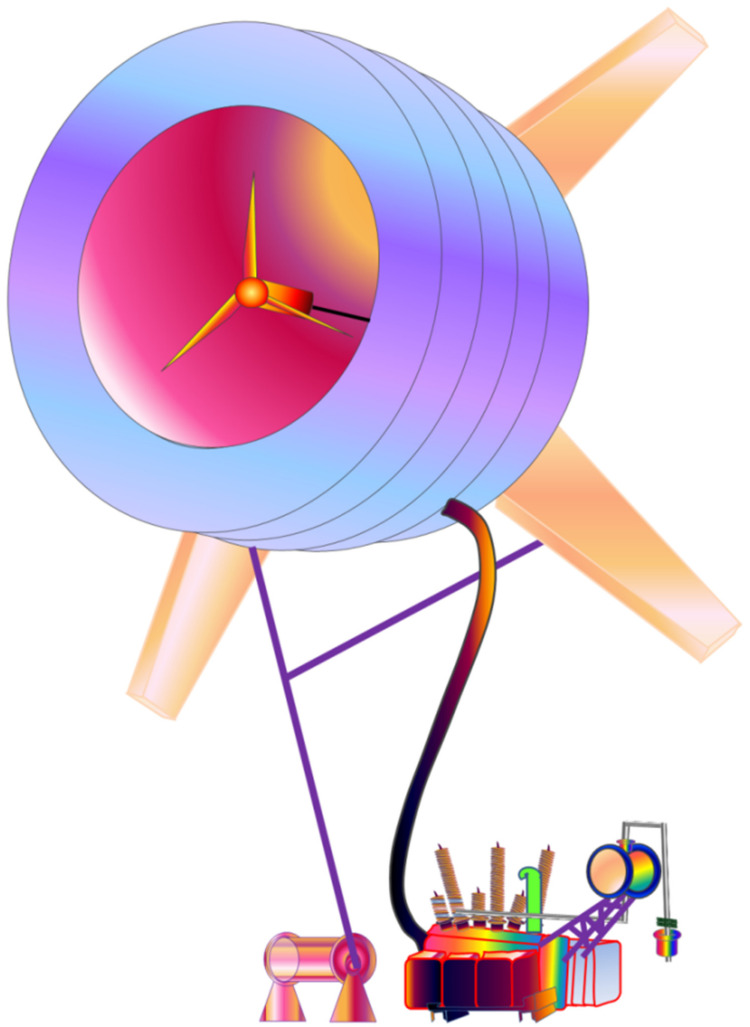
Airborne Wind Turbine [78].

AWE systems are a collective group of devices that harness energy in a similar form as a regular WECS set-up, instead having the external features of an unmanned aircraft or an autonomous kite. An airborne wind turbine is schematically shown in Figure 10 [78]. The system is suspended in air and attached to the ground by means of one or more tethers to keep it in place. This system has some advantages over modern WECS technologies, including a huge reduction in the material needed for construction, since it lacks the requirement of a tower. There have been many concept designs developed for this aspect of power generation, however none of them have been finalized.

The two broad categories of the AWE system involve ground-gen and fly-gen systems. The ground-gen concept aims at converting the mechanical power into electrical power at the ground level, while fly-gen systems do so in the airborne unit itself. The fly-gen systems are further classified into crosswind and non-crosswind, out of which the crosswind turbines have been observed to produce more power [78]. Besides the advantage of lower cost owing to the lesser materials for construction, the airborne wind turbine can be raised high for interacting with high-altitude winds found above 200 m [79]. Simultaneously, the altitudes can be altered to protect it from winds above rated speeds.

However, there are many technical challenges associated that are yet to be overcome for making this usable, such as reliability and durability of flexible components and erosion by wind and water. Recent trends in the research of this system consist of drone concepts, aerodynamic modeling, electronics and sensor technologies for autonomous control of the tethered devices, and controls for autonomous take-off and landing.

#### 6.2.2. Offshore Floating Wind Concepts

This concept differs from traditional offshore concepts by the method of foundation. While the latter employs a fixed foundation to the floor of the ocean, this innovative design is a free-floating structure related to the semi-submersible tension leg or the spar platforms. The floating is controlled by various anchoring systems. Since it is a diversion from the existing fixed off-shore designs, there high potential for the exploration of newer techniques to integrate the tower to the floating base. Moreover, the impact of changing turbine parameters including TSR, blade designs and number of blades on the floating platform can be investigated [80].

**Figure 11:**
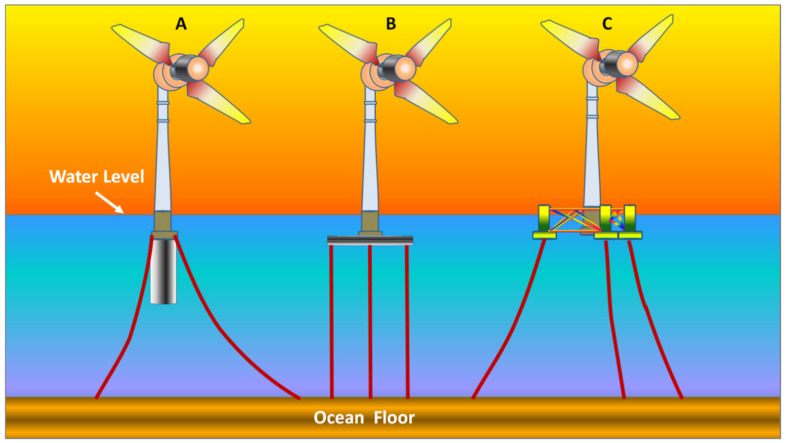
(**A**) Spar-buoy; (**B**) Tension Leg Platform; (**C**) Spar-Submersible [80,81].

Recent progress in this topic has led to evolution of three main types of floating off-shore foundations including spar-buoy, tension leg platform, and spar-submersible, which are depicted in Figure 11A–C, respectively, and is briefly explained below [81]:

*Spar-buoy:* Stability in this set-up is provided by a large cylindrical buoy filled with ballast under the tower structure. This helps in lowering the center of gravity (CG) in the system below the level of the center of buoyancy and increases the stability. Apart from the heavy buoy under the water, the hollow structure above helps to keep the center of buoyancy high.

*Tension Leg Platform*: This design incorporates the buoyant component to be semi-submerged into the ocean surface, which is then attached to the ocean floor by means of tension mooring lines, providing stability to the tower above it. The buoyant structure is maintained; submerged by balancing out the upward buoyant force. it experiences by the mooring lines’ tension.

*Spar-Submersible:* Also called the semi-submersible design, this concept incorporates multiple columns that are linked by bracings or submersible pontoons. The columns provide hydrostatic stability to the structure, whereas the pontoons provide additional buoyancy to it. This system also uses catenary mooring lines to keep it in place [78].

#### 6.2.3. Tip-Rotor Wind Turbine (TRWT)

This concept of wind turbine system integrates high speed, low torque rotors at the tip of the main blades. Therefore, the generator can be coupled directly without the necessity of torque reduction gearbox arrangements for these rotors. The efficiency of these rotors to extract wind energy can go beyond the Betz limit, since it is actually the thrust of these tip-rotors that provides reaction torque to the main rotor of the turbine [82]. The rotation of the bigger main rotor forces the smaller rotors to move and thus harness power. Tests have been conducted in this field by companies making commercial turbines. Siemens had demonstrated a concept of direct drive for e-propellers for an aircraft that could reach performance levels of about 5 kW/kg [83]. Moreover, this signifies that a 10 MW turbine using two blades incorporating this concept, can operate using two generators of 1000 kg each housed at the tip of the blades. This can massively reduce the weight of commercial MW WECS, saving capital costs a huge volume.

#### 6.2.4. Multi-Rotor Wind Turbine (MRWT)

**Figure 12:**
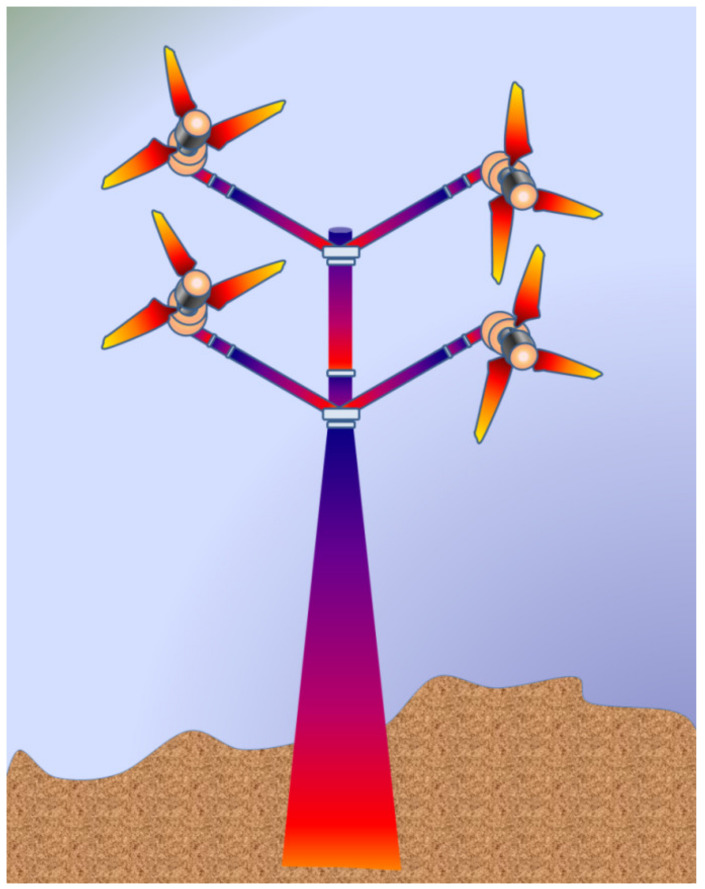
Multi-rotor Wind Turbine [83].

Replacing the large size of a rotor with multiple smaller rotors is helpful to reduce the overall loads on the structure of the turbine. This concept made it possible to install a large power system of about 20-MW at a single site. A company like, Vestas, has adapted this concept to develop a 900 kW four rotor design, illustrated in Figure 12. The most prominent advantage of this technology is the process of standardization as it allows the production process of these small-scale rotors to be industrialized [83]. This process could help is saving production costs in comparison to present large scale rotors, since these large-scale rotors need to be customized for specific application, thus reducing probability of standardization. Studies have enlightened the advantages in maintenance by having smaller components.

The modelling of multi-rotor system needs more development owing to its challenging aspect, as well as aerodynamic studies regarding effects of wake produced by each rotor on adjacent ones need to be looked further, since the design tools for simulating aerodynamic behavior required to visualize a multi-rotor system and turbulent winds are in their elementary stages. The overall design of the setup is also worth looking into for improving the reliability, which is curtailed by the increased static loads and dynamic vibration of increased number of components [84].

#### 6.2.5. Diffuser Augmented Wind Turbine (DAWT)

**Figure 13:**
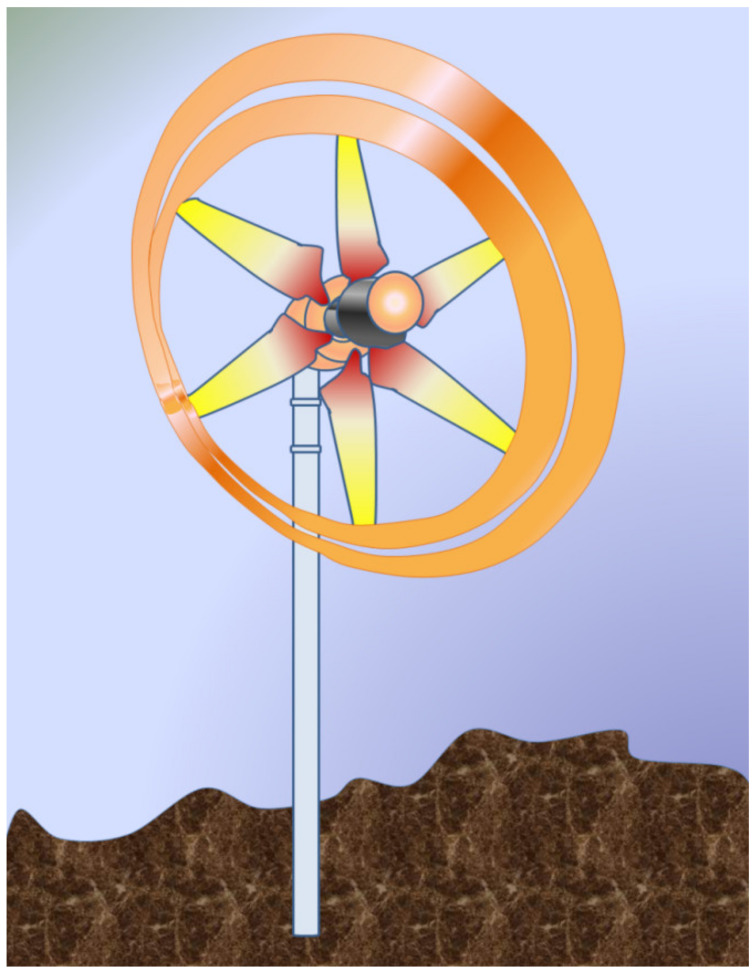
A schematic diagram diffuser augmented wind turbine [86].

Diffuser Augmented Wind Turbines, chiefly abbreviated as DAWT is a modified design of normal horizontal axis wind turbines, with the addition of a diffuser structure around the rotor. The diffuser is encased around with a diffuser that resembles the shape of a funnel, used to concentrate the approaching stream of wind. Modification can be introduced to this structure by adding a rim around the exit side of the diffuser and an inlet shroud at the entrance side (upwind). The diffuser has been proven to increase the generated power compared to traditional HAWTs [85]. A schematic diffuser augmented wind turbine is shown in Figure 13 [86].

The concept of DAWTs is being developed in Japan at a semi-commercial level. The rating of these is in a range of ten kW at present [83], with the most efficient design developed by Kyushu University, who have developed a DAWT system capable of producing 100 kW. This is however the outcome of extensive study and testing of various configurations of DAWTs [86]. This design is an integration of a multi-rotor turbine and a DAWT, effectively making a multi-rotor diffuser augmented turbine. Multi-rotor DAWT systems have been demonstrated to have performance advantage over multi-rotor turbines. Optimization in space between each diffuser can have significant advantage in the overall design extracted power, since the geometric symmetrical structure may not be really advantageous in the intended use, due to flow parameters of free stream.

With a more complex design than its counterpart, it may not be a cheap alternative. However, if it is used properly, it would be suitable in certain situations. At present, diffuser augmented turbines face the biggest challenges of structural loading. Though this design has sound potential, they are best suited for small scale applications.

## 7. Material Aspects

In wind energy conversion systems, both mechanical and electrical components play a vital role. Therefore, the materials used for mechanical and electrical components in the WECS are also extremely crucial. Specifically, the materials used for mechanical parts help to get ‘work’ of the system using a mass to rotate the turbine blade and the materials used for electrical parts help to transmit the mass to a desired location. Typically, a wind turbine is constructed of nearly 25,000 elements, which are grouped into several major components including, tower, nacelle and rotor [87]. The rotor, which contains blades, a hub and a blade pitch system, is attached to a nacelle. The nacelle is connected to the tower. The nacelle comprises numerous electrical and mechanical components, such as main a shaft, a gearbox, generator and control systems. As the turbine blades rotate, it leads to drive an electrical generator through a gearbox. The gear box contains special alloys to adapt wide range of wind speeds [88]. The gear box is placed on top of a tower, which is made of large tubular steel sections. The steel sections are mainly attached to an anchor component and constructed on a foundation [89]. In addition, the wind turbine plant also connected some conventional ground-mounted components including the plant transformer, switchgears, and site cables, which connects between the turbines, to the transformer and to the grid.

**Figure 14:**
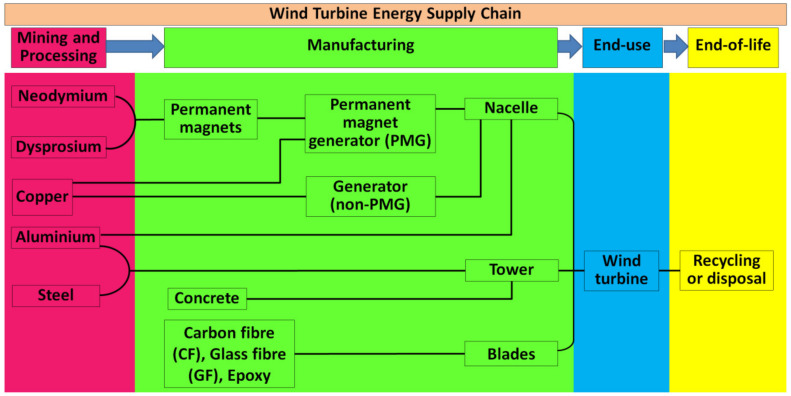
The most useful components used for the wind turbine energy supply chains from the necessary raw materials, processed materials and components, to the final products.

In a rotor subassembly, the rotating body comprises rotor blades, rotor bearings, nose cone, hubs, and the pitch drive system. Therefore, the major mechanical components of the WECS, for which the selection of material is very important, are rotor, main shaft, gearbox, mechanical brakes, nacelle, pitch and yaw drives, and wind measuring equipment. On the other hand, the major electrical components of the WECS, for which the selection of material is very important, are generator, power converter, step-up transformer, and so on. The electrical subsystems convert the mechanical energy into electrical energy from the wind. The most useful components employed for the wind turbine energy supply chains from the necessary raw materials, processed materials, components and up to the final products are depicted in Figure 14 [90].

In the WECS, the wind turbine with efficient wing construction is extremely important to the material scientists. The various components that are used in a wind turbine for renewable energy generation are supportive materials of the turbine, struts, shaft, bearings, chain, sprockets, gear, turbine blades, motor components, tower and so on. In this aspect, NASA invented many turbines with various materials and different technologies, which were mainly aimed at improving energy conversion. The most recent research on wind turbine focused on composite blade materials, steel tube towers, variable-speed generators, and dynamic components including aerodynamics of the blades in horizontal axis turbines, pitch and span control mechanisms. Typically, the weight sharing of an onshore wind turbine plant consists of 75% foundations, 23% turbine, and 2% in cite cables. The turbine part comprises the 59 wt% tower, 22 wt% nacelle, and 19 wt% rotor. The mass distribution of each important elements of wind turbine plant is illustrated in Table 2. Many components, such as tower, nacelle, rotor and foundation are made of iron particularly, mild steel and stainless steel. In steels, a wide range of minor and base metals such as nickel (Ni), chromium (Cr), molybdenum (Mo), and manganese (Mn) are used.

**Figure 15:**
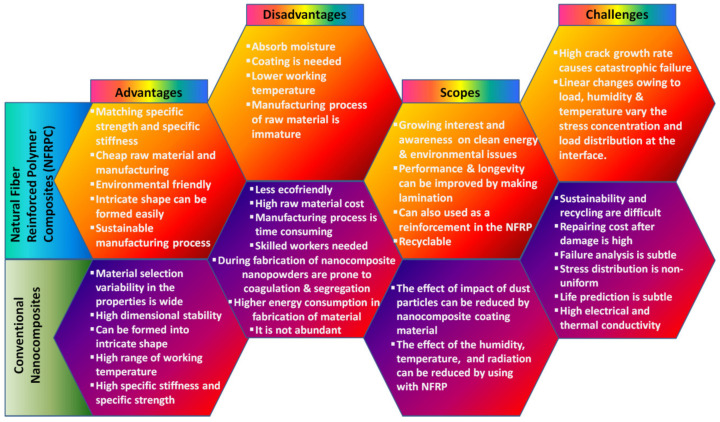
The advantages, disadvantages, scopes and challenges of the conventional as well as natural fiber reinforced composite materials used for the wind turbine blades.

For several years, scientists have been trying to find suitable materials for rotor blades. Earlier the rotor blades were made from heavier metals. These high density or higher mass metals are resistant to environmental conditions such as high turbulent wind, however are rendered not helpful as a result of their large weight. In addition, the different mass concentrations away from the center create larger rotational moment of inertia. This causes a reduction in the acceleration of the rotor. This difficulty led the focus of the material towards composites, which have high rigidity without increasing the mass. Therefore, the advantages, disadvantages, scopes and challenges of the conventional as well as natural fiber reinforced composite materials used for the wind turbine blades are depicted in Figure 15.

In addition, different advanced smart materials such as, piezoelectric materials are trying to use for “wind tree” in order to produce energy or power from the wind flow. The soft flexible materials are used as a leaf element to generate maximum power. The trunk parts of the wind tree-like structure are made of hard materials such as steel. The steel trunks stem into tinier branches, which are attached with many leaf-shaped wind turbines. These wind trees have recently been exploited to all types of wind, from gentle breezes to powerful gusts, in both urban and rural areas [91].

## 8. Conclusions

A long way has been traversed, with an array of technologies implemented in building the modern-day wind energy conversion systems. However, it has long list of prospects in the upcoming future. Shifting the focus away from the carbon-based fossil fuel reserves has opened up a multitude of opportunities waiting to be explored. This review paper discussed in detail the mechanical and electrical components of conventional WECSs, along with the various alterations in the designs, which have helped in categorization of the systems. This review paid much attention to the ecological applications of WECS and wind power plants. It is also to be informed that the hydropower plant could be another alternative sustainable power source since wind flow is a less stable power source. For wind power generation, the water consumption is lowest. It has the lowest relative greenhouse gas emission. Thus, it has the most favorable social impacts and is considered as one of the most sustainable renewable energy sources, followed by hydropower, photovoltaic, and geothermal.

Most commercial applications have adopted HAWT designs for WECS over VAWTs due to the former’s compatibility with large scale power production. Different control techniques and strategies were discussed. A majority of wind turbines in the present context operate on doubly fed induction generators, the control strategies of which have been tabulated expressing the salient features of each design. In the context of the present scenario, the evolving technologies for harnessing power from wind have been highlighted, and the future emerging technologies have also been addressed. The reliability of wind energy has increased exponentially over the last few decades. The expansion of wind energy along with other renewable energy sources will continue to grow in the near future as well.

We have found that materials for the blade and generator are the most critical elements of wind turbines. Since many components of wind power plants use synthetic polymer matrix composite materials, the recycling of composite is a cross-sector challenge. This huge challenge is not only a problem for the wind power plants, but also to its supply chain industries. Therefore, all the sectors connected to synthetic polymer matrix composite materials must look into the matter together in order to develop cost-effective solutions and value chains for the combined volume of composite waste.

## Figures and Tables

**Table 1:** Summary of MPPT control techniques used for DFIG based WECS.

Control Technique	Input Parameter	Prior Knowledge on Wind Turbine	Memory Needed	Complication of Control	Performance of Control
Optimal tip speed ratio (OTSR)	$v_{w}$	Not required	N	↓	++
Wind turbine power curve (WTPC)	$v_{w}$	Required	Y	↓↓	++
Rotational speed of generator Optimal torque (OT)	$\omega_{m}$	Required	N	↓	+
Power signal feedback (PSF)	$\omega_{m}$	Required	Y	↓↓	+
Secure sockets layer (SSL)	$f_{a}$	Required	Y	↓↓↓	+
Hill-climbing search (HCS)	$\omega_{m},\;i_{dc}$	Required	Y	↓↓↓	+

Yes: ‘Y’, No: ‘N’, Low: ‘↓’, Medium: ‘↓↓’, High: ‘↓↓↓’, Good: ‘+’, Very good: ‘++’.

**Table 2:** Mass distribution of each important element of wind turbine plant.

Materials	Mass (%)	Components
Concrete	50–60	Foundation
Steel	25–35	Foundation, tower, nacelle, generator, gear box
Composite materials	2–3	Turbine blades
Electronic components	<1	Generator
Copper (Cu)	<1	Foundation, tower, nacelle, generator, gear box
Aluminium (Al)	<1	Tower, nacelle
Polyvinyl chloride (PVC)	<1	Foundation, Turbine blades
Lubricant and other liquids	<1	Gearbox, generator
Self-cleaning coatings	<1	Turbine blades, tower, gear box

## Data Availability

Not applicable.
